# Multi-stage analysis of *FOXM1, PYROXD1, hTERT, PPARA, PIM3, BMI1 and MCTP1* expression patterns in colorectal cancer 

**Published:** 2022

**Authors:** Samira Shabani, Elahe Elahi, Mandana Bahraniasl, Pegah Babaheidarian, Alireza Sadeghpour, Tayebeh Majidzadeh, Atefeh Talebi, Frouzandeh Mahjoubi

**Affiliations:** 1 *Department of Clinical Genetic, National Institute of Genetic Engineering and Biotechnology (NIGEB), Tehran, Iran *; 2 *Department of Biotechnology, University College of Science, University of Tehran, Tehran, Iran*; 3 *Department of Pathology, Hazrate-Rasoule Akram Hospital, Iran University of Medical Sciences, Tehran, Iran*; 4 * Colorectal Research Centre (CRRC), Hazrate-Rasoule Akram Hospital, Iran University of Medical Sciences, Tehran, Iran*

**Keywords:** Colorectal cancer, Expression pattern, Real-time RT-PCR

## Abstract

**Aim:**

To explore biomarkers with a tumor stage-dependent expression pattern in patients with colorectal cancer (CRC).

**Background:**

The fourth most common cancer in the world is colorectal cancer (CRC). A variation in the gene expression rate is a common change in cancers initiation and the accumulation of these variation changes the behavior of normal cells and turns them into cancer cells.

**Methods:**

Real-time RT-PCR was used to investigate the expression patterns of the *FOXM1*,* PYROXD1*, *hTERT*, *BMI*, *PPARA*, *PIM3* and *MCTP1* genes in 54 patients with stage I to IV CRC and their relation with clinicopathological features of CRC were analyzed. Results: *FOXM1*, *hTERT* and *MCTP1* genes are overexpressed in CRC tumor tissues when compared to normal adjacent tissues in all the stages.

**Results:**

*FOXM1, PYROXD1*,* hTERT, PIM3, BMI1, PPARA* and *MCTP1* had-stage dependent expression. Investigation of the association between clinicopathological features and expression pattern of the studied genes revealed; a) a significant relationship between *FOXM1 *gene expression level and tumor stage, tumor size and lymph node involvement, b) a considerable association between alterations in *PPARA* and *PIM3* expression and lymph node involvement, c) a notable correlation between *hTERT *expression level and the tumor stage and d) a strong correlation between *MCTP1* expression and patient's age only.

**Conclusion:**

Our study indicates that expression profiles of these genes either individually or together can be applied as potential biomarkers for prognosis of CRC.

## Introduction

 The fourth most common cancer in the world is colorectal cancer (CRC) and the worldwide burden of this disease is estimated to rise even further by 2030 (1). The prevalence and percentage mortality of CRC have been found to fluctuate by up to 10-fold globally (2). In Iran the prevalence of this cancer is on the rise and surprisingly, its occurrence in men under 50 years old is remarkable (3, 4).The most important diagnostic factor for this cancer is its stage at the time of diagnosis (5). The traditional method for cancer staging is the tumor-node-metastasis (TNM) classification system (5, 6). Although this classification system provides useful information to physicians, it cannot discriminate between the biological behaviors of different tumors. Many studies consider CRC progress as a stepwise procedure with the accumulation of various genetic alterations (7, 8). Cancer tissue gene expression profiling is anticipated to bring new insights into the underlying causes and understanding of cancer biology as well as improving new methods of prognosis, diagnosis, prediction and therapy. Furthermore, a variation in the gene expression rate is a common change in cancers initiation and the accumulation of these variation changes the behavior of normal cells and turns them into cancer cells. Cancer initiation and growth is tightly controlled by the interaction between genetic and epigenetic factors which leads to differential gene expression. Normal cell growth is blocked with different ways which are critically coordinated alterations in gene expression during carcinogenesis (9, 10). 

**Table 1:** Characteristic features of genes studied in this article

Name/Gene ID	Accession number	Location	Description	Biological activity
*FOXM1*/ 2305	NM_202002.2	12p13	Forkhead box protein M1	Transcription regulation
*PPARA*/ 5465	NM_001001928.2	22q13.31	Peroxisome proliferator-activated receptor alpha	Transcription regulation
*PIM3*/ 415116	NM_001001852.3	22q13	Serine/threonine-protein kinase pim-3	serine/threonine kinase activity
*MCTP1*/ 79772	NM_001297777.1	5q15	Multiple C2 domains, transmembrane 1	calcium-mediated signaling
*PYROXD1*/79912	NM_024854.3	12p12.1	pyridine nucleotide-disulphide oxidoreductase domain 1	oxidoreductase
*hTERT*/ 7015	NM_198253.2	5p15.33	Telomerase reverse transcriptase	Ribonucleoprotein
*BMI1 */ 648	NM_005180.8	10p12.2	BMI1 proto-oncogene, polycomb ring finger	Transcription, Transcription regulation

In the recent research at first CRC patients with different tumor stages were included to study. Then we went through the expression data in literature to elucidate new reported biomarkers that had driven the development of CRC. Previously, a panel of genes by Agendia which is a classifier of robust gene expression (ColoPrint) was identified to significantly improve the prognostic accuracy of pathologic factors in CRC patients with stage II and III.”(11). Therefore, to investigate the relationship between the expression of certain genes and different clinicopathological factors, we selected: a) 4 genes from this panel randomly b) 3 other genes which were not in this panel but were cited a lot in literature. Consequently, we inspected the expression of this panel of genes in Iranian CRC patients, hoping to introduce possible diagnostic or prognostic biomarkers. According to the above description, seven genes (as mentioned in Table 1) were selected. 

## Methods

**Tumor Samples**

This project was permitted by the National Institute for Genetic Engineering and Biotechnology (NIGEB).Colorectal cancer patients were accepted to Rasool e Akram Hospital (a referral governmental hospital) in Tehran (between the years 2010 to 2017). Written consent forms were taken from every case. Fifty four tumor and 48 adjacent normal tissues were prospectively obtained during surgery. The tissue specimens were then stored at -70°C prior to RNA extraction. All patient pathologic information was obtained from the Department of pathology. Colorectal cancer (CRC) tissue staging was carried out in accordance with the TNM classification system (12). 

**RNA purification and cDNA synthesis**

The TriPure Isolation Reagent and RevertAid First Strand cDNA Synthesis Kits were used for RNA purification (Roche Applied Sciences, Germany) and cDNA synthesis (Thermo Fisher Scientific, Germany), respectively .

**Real-time RT-PCR**

Real-Time RT-PCR using the SYBR-Green master mix was carried out by Bosch 's real-time PCR thermal cycler (Roche Applied Sciences, Germany).The amplification process was carried out in a 10 μL reaction volume using 0.1 μM vials, containing 0.5 μM of each primer, 1 μL of cDNA (as template), 5 μL of SYBR-Green master mix, 3 μL of water. 

**Table 2:** Primer sequence details for PCR and Real RT PCR

Amplicon size (bp)	Primer sequence(5´--- 3´)	Gene name
123	For: AGTGTGTACGTGGTCGAG Rev: GGGGATGAAGCGGAGTCT	*FOXM1*
164	GCAGGGGGGAGCCAAAAGGGT TGGGTGGCAGTGATGGCATGG	*PPARA*
172	TGACATTTATTCAAAGTTAAAAGC TAGACACTTTATGCAAACATTTCAA	*MCTP1*
170	AAGAACCTCAACCCTGTGTGGG GGGTCACATCTGTGGGCCTG	*PIM3*
132	TAGACAGATGGGATGGTATGC CCCAGCAGTACAACCTTATAG	*PYROXD1*
228	AGTGTGTACGTGGTCGAG GGGGATGAAGCGGAGTCT	hTERT
200	ATCCTTCTCGTGATGCTGCCA TCATTGCTCGTGGGCATCGT	*BMI1*
219	GCAGGGGGGAGCCAAAAGGGT TGGGTGGCAGTGATGGCATGG	*GAPDH*

The thermal cycle program was as follows: 95°C for 5 min for the initial denaturation step, and an amplification program (95°C for 20, 60°C for 15 and 72°C for 20 seconds, respectively) repeated for 40 cycles. Primers were designed with the oligo7 software (Table 2). The specificity of the primers was theoretically tested by the BLAST database. The glyceraldehyde 3-phosphate dehydrogenase (*GAPDH*) gene was selected as the housekeeping gene.

**Statistical data analysis**

The real-time RT-PCR raw data for each gene was evaluated with the Linreg software. Subsequently, the expression ratio results (sample group difference relative to the control group) for significance and statistical analysis were analyzed with the REST software and SPSS software V22.0 (SPSS, Inc., Chicago, IL). The normality assumption was checked by the Kolmogorov-Smirnov test, and variances between groups were analyzed by the one-way analysis of variance (ANOVA) and independent sample T tests. 

## Results

**Patient pathologic analysis**

In total, 54 CRC tissues and 48 adjacent normal tissues registered at the Rasool-e-Akram Hospital were analyzed in this study (pathological information of 3 patients were absent). The median age of patients at the time of diagnosis was 50.5 years (ranging from 22-79 years). Cases were composed of 26 females and 25 males. The TNM staging information of patients was as follows: 8, 14, 18, and 8 patients were at stage I- IV. Among these samples, 26 were localized to the colon and 19 to the rectum, while for 5 cases the information was not available. Twenty five (% 48) patients were lymph node positive (82% N1 &18% N2) and 29 (%52) were lymph node negative.

**H&E staining**

**Figure 1:**
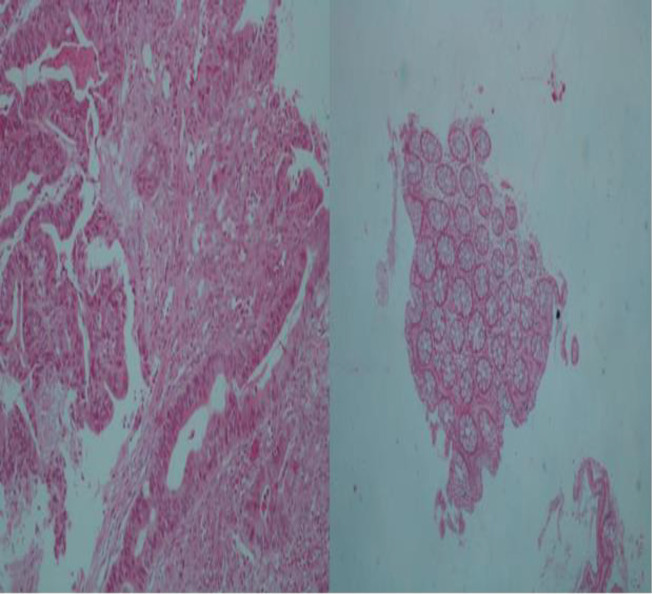
A colorectal tumor cells and B normal colorectal cells characteristics. Hematoxylin –Eosin (H&E) staining verified by pathologist. Magnification (×400)

For pathological purposes, frozen tissues were stained with hematoxylin and eosin (H&E). This was followed by evaluation of tumor content and verification of their histology (Figure 1).

**Expression profiles of** ***FOXM1, hTERT, PPARA, PIM3, PYROXD1, BMI1 and MCTP1*** **in CRC patients from early stage I to advance stage IV.**

***FOXM1***

**Figure 2:**
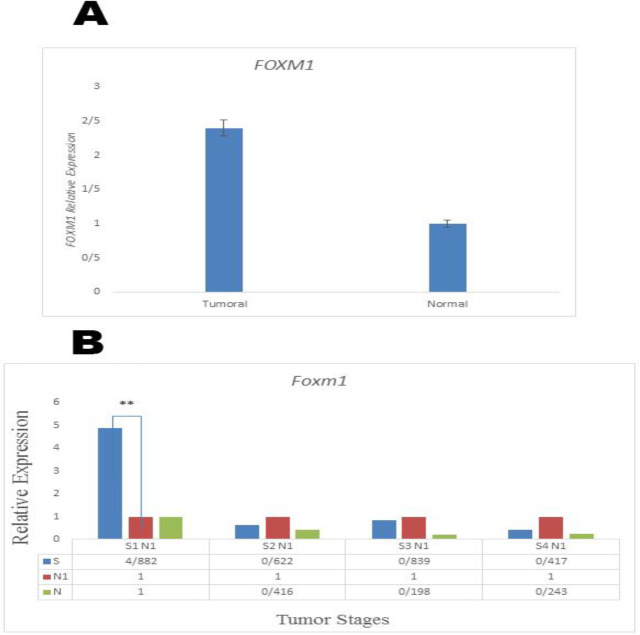
A: Relative expression analysis of *FOXM1* (P ≤ 0.05).B: Stage-dependent expression of *FOXM1* in CRC patients (S1–S4 stands for cancer stages from stage I to progressive stage IV

Relative expression analysis showed that the* FOXM1* gene expression rate was considerably different between tumor and normal tissues in a way that *FOXM1* was upregulated in tumor tissues P (H) = (0.03) (Figure 2). Investigation of the association between clinicopathological features and the *FOXM1* expression level demonstrated a significant association between the grade (stage), tumor size (T), lymph node involvement (N) and the* FOXM1* expression level in our patients (P ≤ 0. 05), (P ≤ 0. 045) (P ≤0.05). No association was detected between the* FOXM1 *expression pattern and age, tumor position, tumor stage, gender and differentiation. *FOXM1* expression analysis at different stages (S1-S4) revealed that *FOXM1* expression increased significantly at early stage I (P≤0.007), and no significant expression was observed at the stages S2, S3, and S4 (Figure 2).

***hTERT***

As expected, *hTERT* gene expression was not detectable in normal tissues while a significant overexpression of *hTERT* was detected in CRC patients. Expression of *hTERT* expression was considerably related to the grade of the tumor (P ≤0.03), and its expression level was significantly amplified in all the grades. No other association was detected between *hTERT *RNA pattern and other clinical characteristics, such as lymph node involvement, age and tumor size.

***PPARA***

**Figure 3:**
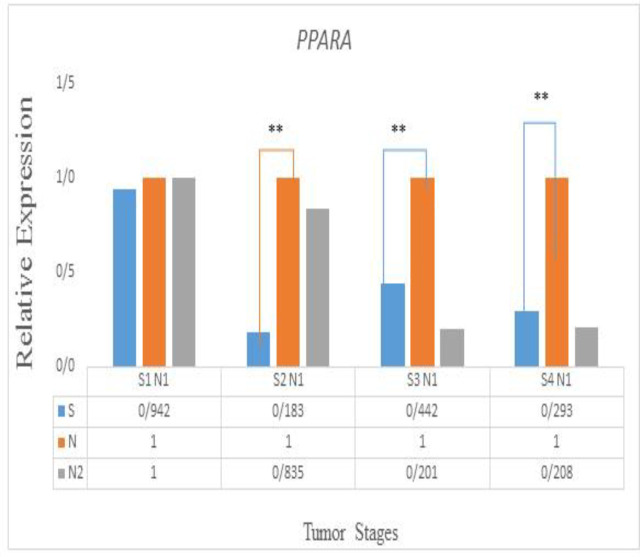
Expression profile of PPARA at different stages (S1-S4)

The *PPARA* expression pattern between tumor and normal adjacent tissues was not considerably different P (H) = (0.7). Interestingly, there was a considerable association between *PPARA* expression level and lymph node involvement in CRC patients (P ≤0.038). The analysis of *PPARA* expression at different stages (S1-S4) indicated that *PPARA* expression decreased significantly in cancer versus control samples in S2, S3 and S4. Furthermore PPARA expression at Stage I was not considerably different (Figure 3).

***PIM3***

**Figure 4:**
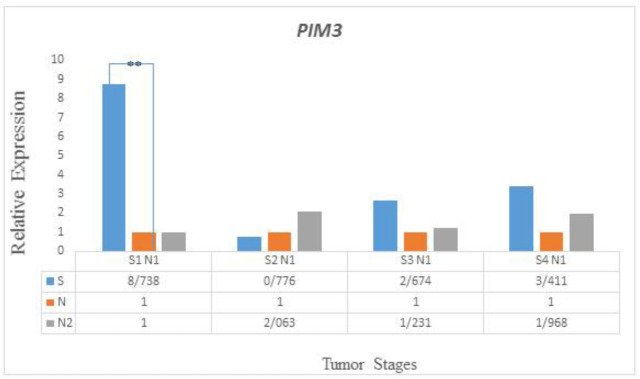
Expression profiles of PIM3 at different stages (S1-S4)

The rate of *PIM3* expression did not increase in tumor tissues in contrast to relative to the adjacent normal tissues P (H) = 0.335. Moreover, a significant association was observed between *PIM3* expression alteration in tumor tissues and lymph node involvement (P ≤.044).The analysis of* PIM3* expression at different stages (S1-S4) showed that *PIM3* expression increased significantly at early stage I (P≤0.001) and no significant expression was found in the S2, S3, and S4 stages (Figure 4).

***PYROXD1***

**Figure 5:**
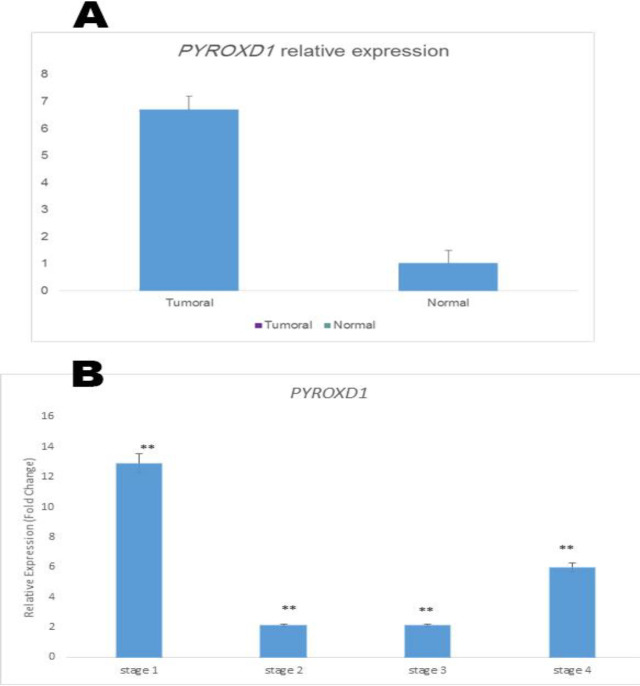
A: Relative expression analysis of PYROXD1 (P ≤ 0.05). A P value less than 0.05 were considered statistically significant

Relative expression result illustrated that *PYROXD1* is up regulated in sample group (in comparison to control group) by a mean factor of 6.780 (P≤0.006). However, no significant association was observed between *PYROXD1* over expression and clinico-pathological features. Additionally, *PYROXD1* expression level was significantly increased in all the tumor stages S1-S4 (Figure 5).

***BMI1***

**Figure 6:**
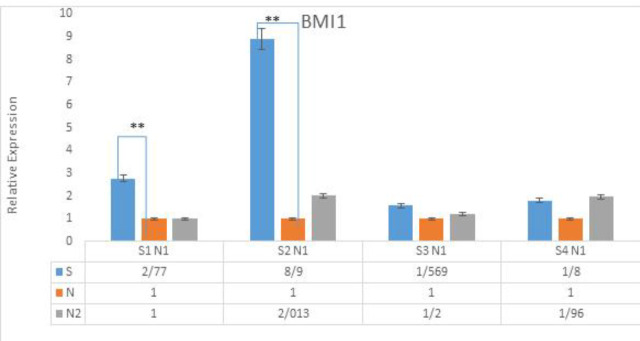
Upregulation of BMI1 in early stages of CRC tumor tissues

The expression rate of *BMI1* did not increase in tumor samples when compared to normal samples (p-value>0.708). No other association was detected between *BMI1* and CRC clinicopathological features. Furthermore*, BMI1* upregulation was identified in both early stage and control samples (Figure 6).

**Figure 7:**
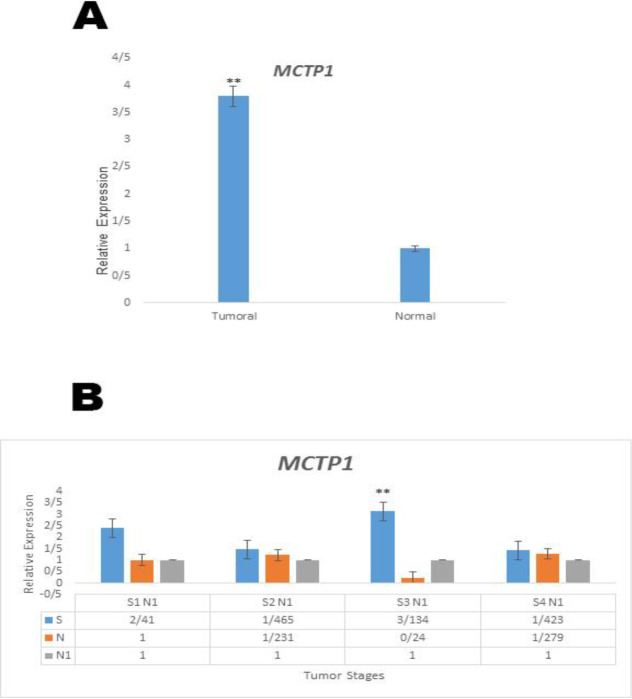
A. Relative expression of MCTP1 (P ≤0.001). B. MCTP1 expression at different stages (S1-S4) of CRC tumor tissue

***MCTP1***

The real-time RT-PCR data were evaluated to deduce the RNA pattern of the MCTP1 gene. There was considerable variation between tumor groups when compared to the control group and MCTP1 was upregulated in tumor groups by a mean factor of 2.844 (P ≤.0.010). Also, variation in expression level of the MCTP1 gene in tumor tissues was strongly correlated with patient’s age (P ≤ 0.018). Furthermore, MCTP1 upregulation was identified amongst advanced-stage and control samples (Figure 7). All the above results are summarized in Table 3 (Table 3).

**Table 3:** Relative expression pattern and the association between clinicopathological features and expression pattern of the selected genes were summarized in this study. A P value less than 0.05 (P ≤0.05) was considered statistically significant

Clinico-pathological features	N (%)	*FOXM1*	*PPARA*	*PYROXD1*	*hTERT*	*BMI1*	*PIM3*	*MCTP1*
Relative expression		0.03** up	0.7	0.006* up	0.05** up	0.708	0.335	0.096
Age ≤50 >50	19 (36) 34 (64)	0.028**	0.746	0.565	0.45	0.71	0.958	0.018**
Gender Male Female	26(49) 27(51)	0.209	0.770	0.318	0.323	0.09	0.659	0.285
TNM stage I II III IV	8 (18) 11(23) 20(42) 9 (18)	0.636	0.747	0.559	0.03**	0.34	0.081	0.567
Tumor Size 5 cm< 5-<8 8-10	19(47) 18(44) 4 (9)	0.045**	0.984	0.075	0.88	0.3	0.297	0.567
Lymph node Involvement N0 N1 N2	20(42) 21(44) 7(14)	0.05**	0.038**	0.668	0.34	0.099	0.04**	0.129
Localization Colon Rectum	24(54) 21(46)	0.165	0.316	0.194	0.3	0.66	0.444	0.865

## Discussion

The fourth most common cancer in the world is CRC, and in Iran, it is much greater than the global average, with a prevalence of 160 out of every 100,000 people (13, 14).The most important diagnostic factor for this cancer is cancer stage at the time of diagnosis. The traditional method for cancer staging is based on the tumor-node-metastasis (TNM) system. Although this method provides effective clinical information of tumor's stage or grade, but, unfortunately, it is unable to give a precise biological classification, and more importantly cannot discriminate between the biological behavior of various tumors (15). Accordingly, it is essential to find an accurate and reliable method that can improve individual treatment.Numerous studies have compared CRC gene expression pattern in normal and cancer tissues at different stages of the disease (16, 17).Herein, we investigated the expression pattern of the selected genes in CRC patients to identify biomarkers which discriminate among colorectal cancers with altered stages. The purpose of this research was to identify biomarkers with a tumor stage-dependent expression pattern so as to develop cancer staging procedures and explore the regulatory mechanisms of CRC (18). 

The Forkhead box protein M1 transcription factor* (FOXM1) * has been shown to have a crucial function in cell cycle progress, and in the S and G2/M phases it has exhibited extreme expression (19, 20). Recently, a growing number of studies have described *FOXM1* as a key oncogenic transcription factor as it can promote tumor progression (21). Emerging data has shown that *FOXM1* regulates gene expression essential to proliferation, apoptosis, and cell-cycle progression, thereby signifying its overall function in tumor growth (22).Several researches have confirmed that *FOXM1* is overexpressed in different cancers and this elevated expression has a vital role in cancer development (23, 24). Furthermore, it was previously been reported that *FOXM1* overexpression is related to the presence of the progressive TNM stage and metastasis lymph node, suggesting that *FOXM1* is possibly involved in cancer metastasis and invasion (24-27). In our research, FOXM1 overexpression was also significantly detected in tumor specimens (P ≤ 0. 03). In the current study, it was revealed that the *FOXM1* expression pattern was clearly related to grade, lymph node involvement and tumor size in CRC (P ≤0.05, P ≤ 0. 045, P ≤0.05, respectively). Analysis of *FOXM1* expression at different stages (S1-S4) revealed that its expression increased significantly at early stage I (P≤0.007), with no significant expression being observed at the S2, S3, and S4 stages. One possible interpretation is that since *FOXM1* is a transcription factor, its high level of expression in the initial phases of tumor development can alter cell proliferation and cell-cycle progression, thereby aiding tumor formation.

The Nuclear receptor subfamily 1 group C member 1 protein (NR1C1) also well-known as the peroxisome proliferator-activated receptor alpha (PPARα) is a nuclear protein (28)*, *which belongs to the subfamily of peroxisome proliferator-activated receptors. The fatty acid products and their derivatives are mediated by these receptors at the transcriptional level. Regarding the regulatory role of *PPARs* in lipid metabolism, these receptors control cell survival, proliferation and differentiation through these pathways, thus monitoring tumorigenesis in different tissues (29, 30). A large number of studies have shown that *PPARα* targets more than a hundred genes (18, 31, 32). To date, *PPARα* pattern in colorectal malignancy has not been studied. Accordingly, the RNA pattern of *PPARα* in CRC, and the association of *PPARα* expression and the patients' clinicopathological features was investigated in CRC. Our result showed that the RNA pattern of *PPARA* was not considerably altered in tumor and normal parallel tissues (P ≤0.05). Interestingly, there was a considerable association between the *PPARA* expression level and lymph node involvement in CRC patients (P ≤0.038). These data suggest that alteration in *PPARA* expression level may be involved in colorectal tumor invasion. The analysis of* PPARA* `expression at different stages (S1-S4) indicated that its expression decreased significantly in cancer versus control samples at the S2, S3 and S4 stages. Furthermore, *PPARA* expression at Stage I was not considerably different. In this study, in contrast to the robust gene expression classifier (ColoPrint) (33), the *PPARA* expression level was not only considerably different between tumor and normal adjacent tissues, but its expression level was also found to decrease in certain stages of CRC. 

Human telomerase reverse transcriptase (encoded by the *hTERT* gene) is crucial for the replication of chromosome ends (34). Furthermore, *hTERT* has various molecular functions and is involved in numerous essential biological processes (http://www.uniprot.org/uniprot/O14746). It has been found that *hTERT* expression increases in various human cancers (35). As expected, *hTERT *gene expression was not detectable in normal tissues while a significant overexpression of *hTERT* was detected in CRC patients. The *hTERT* RNA level was considerably related to cancer grade (P ≤0.03). Its level was noticeably amplified in all the grades. There was not any association between *hTERT* pattern and clinical features. One possible interpretation for this finding is that in the primary stages involving precancerous lesions, most tumors go through constant telomere shortening, thereby activating the telomerase enzyme which leads to tumor progression.

Pyridine nucleotide disulphide oxidoreductase domain 1 (*PYROXD1*) gene belongs to flavoprotein family and catalyze the pyridine-nucleotide-dependent reduction of thiol residues in proteins. (36). One of the cause of chronic inflammation is oxidative stress and the activation of chronic inflammation pathways mediates most chronic diseases and cancers(37, 38).The role of *PYROXD1* in cancer biology and other disease has not yet understood. For the first time we studied *PYROXD1* expression level in colorectal cancer and we showed that *PYROXD1* is up regulated in this cancer. However, no significant association was observed between *PYROXD1* over expression and clinicopathological features. Additionally, *PYROXD1* expression level was significantly increased in all the tumor stages S1-S4. The consistent expression of *PYROXD1* across all the cancer stages may indicate that *PYROXD1* gene contribute in many major biological pathways involved in cancer formation and progression.

The *PIM3* gene codes for the provirus integrating site moloney murine leukemia virus (Pim) family of proteins which have serine ⁄threonine kinase activity (39, 40). Literature review introduces *PIM3 *as a proto-oncogene which can prevent apoptosis and help tumorgenesis by delivering survival signaling and inducing the release of anti-apoptotic proteins (41-43). In this study, *PIM3* was not overexpressed in cancerous tissues of CRC patients. Moreover, a significant association was observed between alteration in *PIM3* expression in tumor tissues and lymph node involvement.The analysis of* PIM3* expression at different stages (S1-S4) showed that *PIM3* expression increased significantly in early stage I (P≤0.001) and no significant expression was found at stages S2, S3, and S4. In this study, although *PIM3* expression was generally not considerably different between tumor and normal adjacent tissues, but it was found that *PIM3* was significantly expressed at early stage I. Regarding the aberrant expression of *PIM3* and its function as a proto-oncogene in various cancers, it seems that in CRC, *PIM3* has stage-dependent expression and in stage I of CRC, *PIM3 acts* as a proto-oncogene, helping tumor formation. However, *PIM3* expression was found to be not significant in the other stages of cancer. 

The *BMI1* gene encodes a ring finger protein that belongs to the Polycomb group  (PcG) (44), which has a critical function in maintaining proliferation, cell differentiation, regulating cellular memories and stem cell self-renewal (45, 46). The existing literature suggests a significant *BMI1* function in malignancy and its upregulation in different cancers (47, 48). In this study, although* BMI1* was not upregulated in tumor tissues when compared to adjacent normal tissues, however, its upregulation was observed at stages I and II of CRC relative to the control samples. These results suggest that *BMI1* also has stage-dependent expression, and thus, these data support the biological role of *BMI1* in CRC, especially at stages I and II. 

The multiple C2 domain and transmembrane protein 1 (encoded by the *MCTP1* gene) is composed of multiple C2 domains and binds to calcium in the absence of phospholipids via the C2 domains (49). It is involved in calcium signaling, with calcium acting as a secondary messenger. Calcium signaling has important roles in a wide range of physiological processes including cell growth and proliferation, enzyme activity, permeability of ion channels and other processes (50, 51). Real-time PCR revealed a considerable alteration in sample cases relative to the control cases. Variation in the expression level of the *MCTP1* gene in tumor tissues was strongly correlated with patient’s age. Furthermore*, MCTP1* upregulation was identified in advanced-stage tissues when compared to the control samples.

While only the *FOXM1*, *PYROXD1*, *hTERT* and *MCTP1* genes were overexpressed in CRC tumor tissues relative to the normal adjacent tissues at all the stages, but *FOXM1****, ****PYROXD1, hTERT, PIM3, BMI1, PPARA and MCTP1* were found to have stage-dependent expression. The *FOXM1*,* hTERT*,* MCTP1*,* BMI1* genes were involved in cell growth,* PIM3* was involved in cell death, and *PYROXD1* was involved in oxidative stress. We hope such efforts of using molecular staging signatures may develop cancer staging procedures by introducing potential biomarkers and significantly benefit the development of personalized medicine in CRC.

## Conflict of interests

The authors declare that they have no conflict of interest.
